# *Gammarinema scyllae* sp. n. and *Monhystrium mangrovi* sp. n. (Nematoda: Monhysteridae) from land crabs from New Caledonia

**DOI:** 10.1007/s11230-021-10017-1

**Published:** 2021-12-18

**Authors:** Rickard Westerman, Mohammed Ahmed, Oleksandr Holovachov

**Affiliations:** 1 Department of Zoology, Stockholm University, Stockholm, Sweden; 2 Department of Zoology, Swedish Museum of Natural History, Stockholm, Sweden

## Abstract

Two new species of nematodes associated with crabs are described from New Caledonia. *Gammarinema scyllae* **sp. n.** from the gill chambers of *Scylla serrata* (Forsskål) is characterised by 3–4 mm long body, small outer labial and cephalic sensilla, distinct ocelli, short straight spicules and sub-cylindrical tail. *Monhystrium mangrovi* **sp. n.** from the gill chambers and body cavity of mangrove crab *Neosarmatium* sp. is characterised by 1–1.4 mm long body; outer labial sensilla longer than cephalic sensilla, amphid located at level with posterior stoma chamber, denticles in posterior stoma chamber and five pairs of genital papilla on tail. Phylogenetic relationships of two new species and other nematodes from the family Monhysteridae are analysed based on 18S and partial 28S rDNA sequences.

## Introduction

The family Monhysteridae is predominantly free-living taxon with over 180 known species (Fonseca & Decraemer, 2008), that includes several small genera that live in close association with other organisms. These include the genera *Gammarinema *Kinne & Gerlach, 1953, *Monhystrium *Cobb, 1920*, Tripylium *Cobb, 1920, *Testudinema* Abebe et al., 2012 and *Odontobius *Roussel del Vauzème, 1834, as well as few species from the genus *Halomonhystera *Andrássy, 2006*.*

*Gammarinema* is a rather small genus, containing species associated decapod and peracarid crustaceans found in both limnic and marine environments. There are only eight known species: *Gammarinema ampullocauda* (Paramonov, 1926) Lorenzen, 1986, *G. cambari* (Allen, 1933) Osche, 1955, *G. gammari* Kinne & Gerlach, 1953, *G. ligiae* Gerlach, 1967, *G. cardisomae* Riemann, 1968, *G. paratelphusi* (Farooqui, 1967) Sudhaus, 1974, *G. mesidoteae* Belogurov, Kulikov & Russkikh, 1978 and *G. prilepskyi *Tchesunov & Pletnikova, 1986. Of these, *Gammarinema ampullocauda* was found free-living in a small lake on the Kinburn Split near the Black Sea (Paramonov, 1926); *G. cambari* inhabits the gill-chambers of *Cambarus acuminatus* Faxon and *Procambarus blandingii* (Harlan), two limnetic decapod species found in the USA (Allen, 1933); *G. cardisomae* is also found in gill chambers but of a different decapod species, *Cardisoma guanhumi* Latreille, from marine supralittoral habitats in the Caribbean (Riemann, 1968). Another species, *G. gammari* has been found in several different amphipod species, such as *Gammarus locusta* (Linnaeus)*, G. oceanicus* Segerstråle*, G. salinus* Spooner and *G. zaddachi* Sexton*,* all marine and living in the Baltic and Bering Seas (Kinne & Gerlach, 1953; Tchesunov & Pletnikova, 1986). Another European species, *Gammarinema ligiae* was found on an isopod *Ligia oceanica* (Linnaeus) living in a marine supralittoral habitat in the Baltic and Helgoland (Gerlach, 1967). The sixth species, *G. paratelphusi* was found in the gill chambers of a limnetic decapod *Paratelphusa* sp. from Maharashtra, India (Farooqui, 1967). The last two species *G. mesidoteae* and *G. prilepskyi* were discovered on an isopod *Mesidotea entomon* (Linnaeus) from the Pacific coast of Russia and from Barents Sea (Tchesunov & Pletnikova, 1986).

*Monhystrium *includes only five known species: *M. wilsoni* (Baylis, 1915) Cobb, 1920,* M. transitans* Cobb, 1920,* M. inquilinus* Riemann, 1969,* M.* *brevis *Yoshimura, 1990 and* M*. *tenuis *Yoshimura, 1990 which are found exclusively in gill chambers of land crabs in different parts of the world. The first species was discovered in 1910 in the gills of a purple land crab *Gecarcinus ruricola* (Linnaeus) in Jamaica (Baylis, 1915). The second species was described in 1920 by N.A Cobb from the gill chambers of the same purple land crab and subsequently also from the gills of the blackback land crab *G. lateralis* (Guérin) from Jamaica (Cobb, 1920). In 1969 F. Riemann described the third species of *Monhystrium*, *M. inquilinus*; this new species was also found in the gill chambers of a land crab, but this time in a blue land crab *Cardisoma guanhumi* caught in Colombia (Riemann, 1969). The last two species to be added to the genus were *Monhystrium tenuis* and *M. brevis,* described by K. Yoshimura (1990); *M. tenuis* was found in the gills of *Parasesarma plicatum* (Latreille),* P. pictum* (De Haan) and *Clistocoeloma merguiense* de Man while *M. brevis* was found in the gill chamber of the red-clawed crab *Chiromantes haematocheir* (De Haan)*, C. dehaani* (H. Milne Edwards) and *Chasmagnathus convexus* (De Haan) and also in the gills of flower crabs *Orisarma intermedium* (De Haan).

## Materials and Methods

*Sampling and specimen preparation.* Nematodes were found during parasitological inspection of land crab specimens obtained from the local inhabitants. Nematodes were carefully removed from the gills of crabs and immediately preserved in 5% formaldehyde solution, 95% ethanol solution and RNAlater. For light microscopy, specimens were transferred to pure glycerine using Seinhorst’s (1959) rapid method as modified by De Grisse (1969). Permanent nematode mounts on glass slides were prepared using the paraffin wax ring method. All curved structures were measured along the curved median line. Terminology follows Maggenti et al. (2005). Abbreviations are according to Hunt & Palomares-Ruis (2012).

*Molecular analysis.* DNA extraction was performed on two individuals for each species. Individual nematodes were each placed in 1.5 ml microcentrifuge tubes containing 20 μl buffer ATL (Qiagen, Sweden) and stored at -20°C until all samples were ready for extraction. During the extraction, 160 μl of buffer ATL was added to each sample. This was followed by the addition of 20 μl proteinase K, vortexing and incubation in an incubating microplate shaker at 56°C and 300 rpm overnight. The lysed samples were further processed to obtain pure DNA following the manufacturer’s instructions for genomic DNA extraction using the Qiagen QiAmp DNA Micro kit. Two regions of the rDNA gene, the nearly full-length of the 18S and the D2–D3 expansion segment of 28S, were amplified. The approximately 1800 bp region of the 18S rRNA gene was amplified as two overlapping fragments using the primer sets 988F–1912R for the first fragment and 1813F–2646R for the second fragment (Holterman et al., 2006). Polymerase chain reaction (PCR) for both fragments was performed in 25 μl reaction mix using Illustra Hot Start Mix RTG 0.2 ml reaction kit (GE Healthcare Life Sciences, Sweden). The reaction mix consisted of 1 μl (0.4 μM) of each primer, 2 μl template DNA and 21 μl nuclease-free water. The reaction conditions were 5 min at 95°C; 5 cycles of (30 sec at 94°C, 30 sec at 45°C and 30 sec at 72°C); 35 cycles of (30 sec at 94°C, 30 sec at 54°C and 30 sec at 72°C); and a final extension for 5 min at 72°C. The D2–D3 segment of the 28S rRNA gene was amplified using the primers D2Af and D3Br (Nunn, 1992). PCR was performed in 25 μl reaction mix containing 1 μl (0.4 μM) of each primer, 2 μl template DNA and 21 μl nuclease-free water. The PCR conditions were 4 min at 94°C; 35 cycles of (94°C for 60 sec, 54°C for 90 sec and 72°C for 2 min); final extension for 10 min at 72°C. Enzymatic PCR clean-up was performed on the PCR product using Exonuclease I and Shrimp Alkaline Phosphatase (New England Biolabs, MA, USA). The purified PCR products were sent out to Macrogen Europe B.V. (Amsterdam, the Netherlands) for sequencing. Each amplicon was sequenced in both directions using the forward and reverse PCR primers. The trace files of the individual sequences were visualized inside BioEdit (Hall, 1999) and trimmed to high quality. The trimmed forward and reverse sequences were then assembled using Fragment Merger online tool (Bell & Kramvis, 2013). The two fragments of the 18S rRNA gene were also assembled into contigs using the Fragment Merger online tool.

*Phylogenetic analysis.* Alignment from Ahmed and Holovachov (2020) for 18S rRNA gene was used as template for alignment and annotation. New sequences were aligned to a fixed template alignment using AliView (Larsson, 2014). Partial 28S rDNA sequences were aligned de novo in AliView. Phylogenetic trees were built using RAxML ver. HPC2 (Stamatakis, 2014) via the CIPRES portal (Miller et al., 2010) for the Maximum Likelihood inference of the partitioned dataset. The GTR nucleotide substitution model was used for non-paired sites, whereas the RNA7A (Higgs, 2000) substitution model was used for paired sites. Bootstrap ML analysis was performed using the rapid bootstrapping option with 1000 iterations.

**Monhysteridae de Man, 1876**

*Gammarinema* Kinne & Gerlach, 1953

- *Type species:*
- *Gammarinema gammari* Kinne & Gerlach, 1953, by original designation.
- *Other species:*
- *Gammarinema ampullocauda* (Paramonov, 1926) Lorenzen, 1986
- = *Monhystera ampullocauda *Paramonov, 1926
- *Gammarinema cambari* (Allen, 1933) Osche, 1955
- = *Rhabditis cambari *Allen, 1933
- *Gammarinema ligiae* Gerlach, 1967
- *Gammarinema paratelphusi* (Farooqui, 1967) Sudhaus, 1974
- = *Branchinema paratelphusi* Farooqui, 1967
- *Gammarinema cardisomae* Riemann, 1968
- *Gammarinema mesidoteae* Belogurov, Kulikov & Russkikh, 1978
- *Gammarinema prilepskyi *Tchesunov & Pletnikova, 1986
- *Gammarinema scyllae* **sp. n.**

*Genus diagnosis:* Body length medium to long (0.7–4.0 mm). Inner labial sensilla papilliform; outer labial and cephalic sensilla in one circle, same or different in length, with either outer labial or cephalic sensilla being longer than the others. Amphids round; less than 1.5 head diameters from the anterior body end. Ocelli present or absent. Buccal cavity funnel shaped, with weakly to moderately cuticularized anterior chamber of stegostom, and narrow posterior chamber of stegostom; posterior chamber with 3 or 6 denticles or without any. Pharynx cylindrical. Progaster present. Ventral gland well-developed and visible, excretory pore opens along the anterior region of the pharynx or within the labial region. Ovary and testis on the right-hand side of intestine. Spicules simple and narrow, straight to arcuate. Gubernaculum platelike, with or without apophysis. Precloacal supplements may be present. Tail conoid to subcylindrical. Caudal glands opening via common spinneret. Usually found associated with crustaceans.

Gammarinema scyllae sp. n.

*Type host and locality:* The nematodes were found in the gill chambers of the mud crab *Scylla serrata* (Forskål) collected near Wagap, Poindimié commune, Northern Province, New Caledonia (HYNC4666; July 12, 2018).

*Type material:* Holotype male and two males and seven females paratypes on slides MNHN-BN514–MNHN-BN518 are deposited in the meiofauna collection of the National Museum of Natural History in Paris, France (MNHN). Seven males, six females and 73 juveniles paratypes on slides SMNH Type-9353–SMNH Type-9359 are deposited in the Invertebrate type collection of the Department of Zoology, Swedish Museum of Natural History, Stockholm, Sweden.

*Etymology*: The species name scyllae is derived from its host name *Scylla*.

*ZooBank registration*: urn:lsid:zoobank.org:pub:A0F29FA2-B06B-4CFF-A100-BE4F292E8188 (publication); urn:lsid:zoobank.org:act:CAA34FB3-AD20-43B8-B3B6-73D1A64914F9 (species).

*GenBank acc. numbers*: Sequences obtained are deposited in GenBank under the accession numbers MZ274175 and MZ274176 for the D2-D3 segment of the 28S rRNA gene and MZ274171 and MZ274172 for the nearly full-length 18S rRNA gene.

### Description

*Diagnosis: Gammarinema scyllae* **sp. n.** is characterised by 3–4 mm long body, small outer labial and cephalic sensilla, distinct ocelli, short straight spicules and sub-cylindrical tail. In gill chambers of the mud crab *Scylla serrata*.

**Fig. 1:**
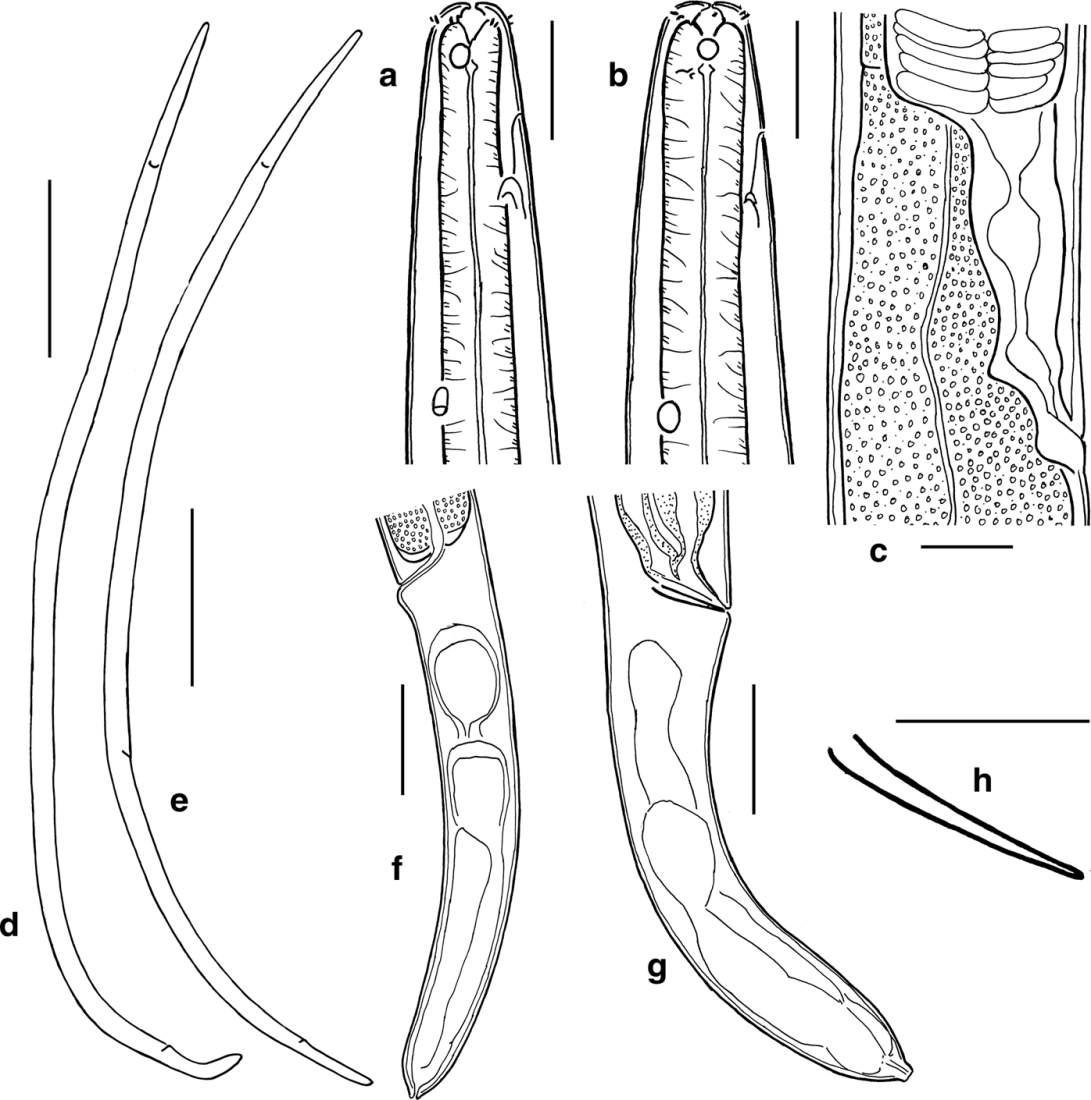
*Gammarinema scyllae* **sp. n.** a. Female anterior end; b. Male anterior end; c. Vulval region; d. Entire male; e, Entire female; f, Female tail; g. Male tail; h. Spicule. Scale bars: a–c, h = 20 µm, d-e = 500 µm, f–g = 50 µm.

**Fig. 2:**
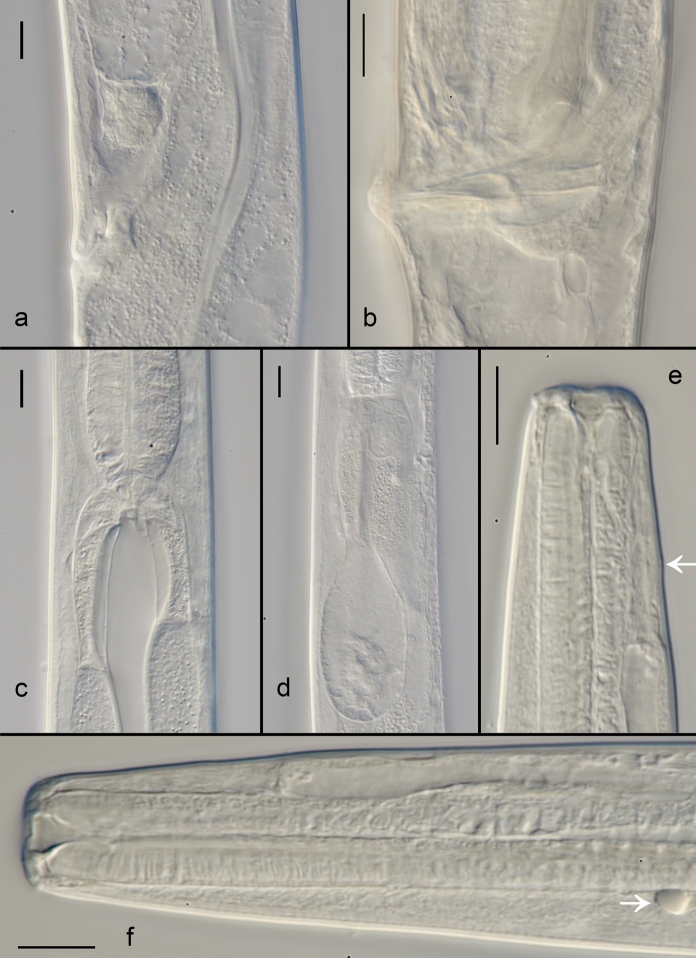
*Gammarinema scyllae* **sp. n.** Female vulval region; b. Male cloacal region / spicules; c. Pharyngo-intestinal junction; d. Renette cell; e. Excretory pore (arrow); f. Anterior region showing ocellus (arrow). Scale bars: a–c, f = 10 µm.

**Fig. 3:**
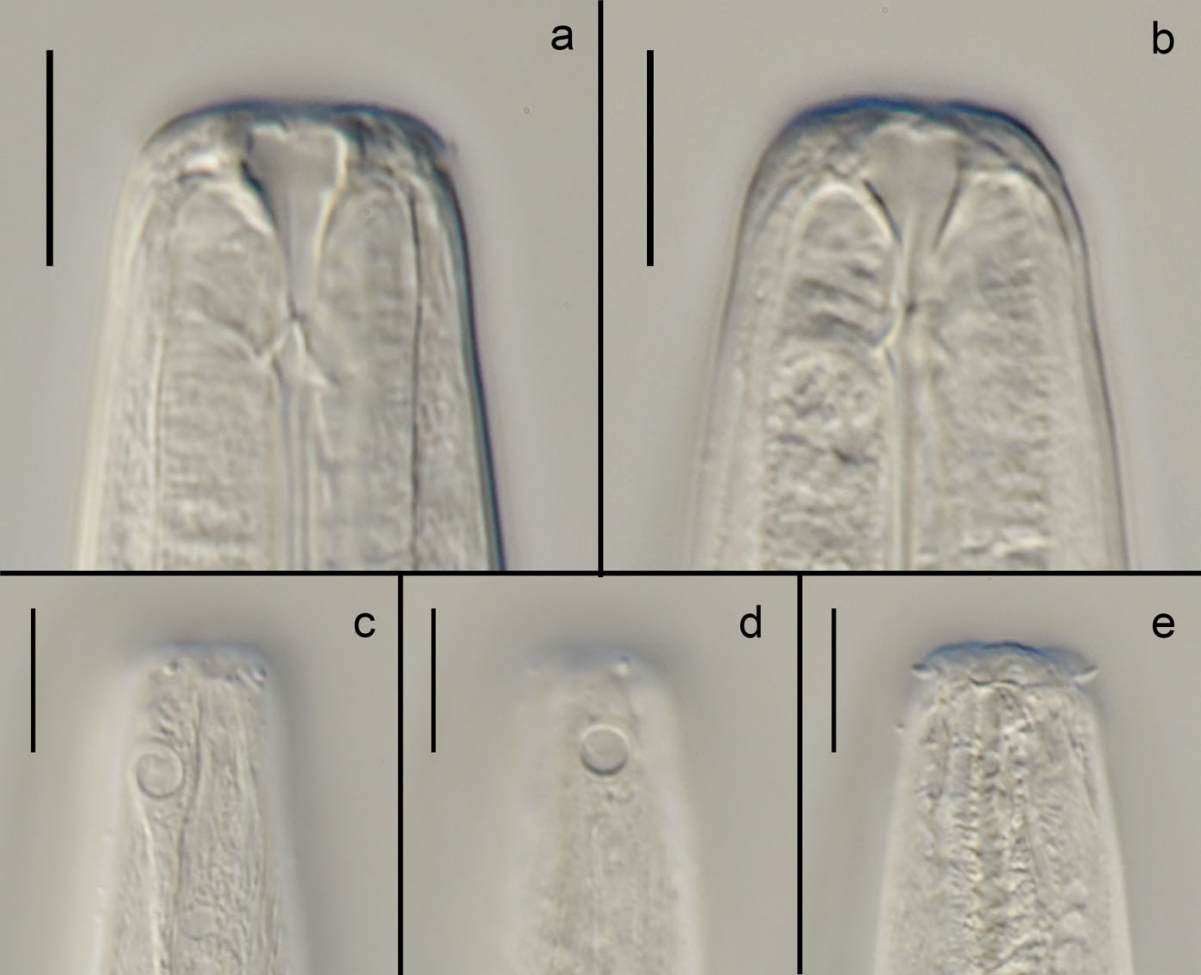
*Gammarinema scyllae* **sp. n.** a–b. Anterior end, median section; c–d. Amphid; e. Anterior sensilla. Scale bars: a–e = 10 µm.

*Adult.* (Figures 1, 2, 3, Table 1). Body cylindrical, ventrally curved upon fixation in females and in males, tapering slightly towards both extremities along pharyngeal region and on tail. Cuticle smooth. Somatic sensilla present, small setiform. Body pores absent. Lateral alae absent. Cephalic region flattened. Six equal lips surrounding mouth opening. Inner labial sensilla small, located on anterior surface of lips. Outer labial sensilla small setiform, located at the base of lip region. Cephalic sensilla papilliform, located at the same level as labial sensilla. Amphideal opening round, located at level of stoma base. Ocelli present. Buccal cavity conoid in general, cheilostom and gymnostom both cylindrical and very short; stegostom funnel-shaped (conoid anteriorly and tubular posteriorly), with weakly cuticularised walls. Posterior chamber poorly defined, denticles indistinct, edges of pharyngeal radii visible. Pharynx uniformly muscularized along its entire length, gradually widening posteriorly but without any valves or bulbs. Cardia small and conical. Intestinal lumen well developed, progaster present. Secretory-excretory system and secretory-excretory pore present, renette cell located along anterior part of intestine, excretory pore located about 1.2 times labial region diameter from the anterior end. Tail cylindrical, with broadly rounded terminus. Caudal glands present, opening towards extension via a common spinneret. Caudal gland cells located in the tail. Spinneret not cuticularised.

**Table 1:** Measurements of *Gammarinema scyllae* **sp. n.** All measurements in µm

Characters	holotype male	4 males	9 females
Body length	3360	3245 ± 177 (2989–3367)	3357 ± 257 (2968–3738)
Body diameter	59	57 ± 4 (51–59)	57 ± 5 (49–63)
Pharynx length	424	418 ± 4 (414–424)	422 ± 26 (376–462)
Tail length	245	232 ± 10 (222–245)	237 ± 22 (201–270)
Anal body diameter	47	46 ± 2 (44–47)	41 ± 2 (37–44)
a	57.0	59.4 ± 6.6 (54.7–76.3)	57.0 ± 0.9 (56.1–58.3)
b	7.9	8.0 ± 0.5 (7.1–8.7)	7.8 ± 0.4 (7.2–8.1)
c	13.7	14.0 ± 0.8 (13.2–15.1)	12.6 ± 1.8 (10.8–15.1)
c´	5.2	5.1 ± 0.3 (4.7–5.4)	5.8 ± 0.7 (4.7–6.3)
V	–	–	62.4 ± 0.9 (61.4–64.6)
Labial region diameter	14	13.3 ± 1.0 (12.5–14.5)	14.1 ± 0.9 (13–16)
Cephalic setae length	2	2.2 ± 0.3 (2–3)	1.9 ± 0.7 (1.5–3.5)
Amphid diameter	3.5	3.6 ± 0.3 (3.5–4)	3.3 ± 0.3 (3–3.5)
Amphid from anterior end	8	7.4 ± 1.0 (6–8)	7.1 ± 0.6 (6–8)
Stoma length	1	12.4 ± 0.6 (12–13)	13.0 ± 1.2 (11–15)
Ocelli from anterior end	68/80	72 ± 8 (63–84)	72 ± 11 (56–96)
Excretory pore from anterior end	17	18.9 ± 2.1 (16.5–21)	16.3 ± 4.0 (12–19)
Vulva length	–	–	17.9 ± 2.4 (14–22)
Rectum length	–	–	34 ± 7 (28–42)
Spicule length	35	34 ± 2 (32–36)	–

*Female*. Reproductive system monodelphic. Ovary branch outstretched, extends anterior but not reaching the cardia, on the right-hand side of intestine. Post-vulval uterine sac absent. Vagina directed anteriorly. Vulva located posterior to midbody, a transverse ventral slit, not cuticularized.

*Male.* Reproductive system monorchic, on the right-hand side of intestine. Spicules paired and symmetrical, straight and relatively thin conoid, equal to 0.7–0.8 anal body diameters in length. No pre- or post- cloacal sensilla or supplements.

*rRNA.* Sequences include two nearly full length 18S rRNA gene and two partial 28S rRNA gene representing D2D3 domain. Sequence variability of the 18S rRNA gene was small, less 1–7 bases difference; sequences of D2D3 domain of 28S rRNA gene were identical. This is the first species from the genus *Gammarinema* to be sequenced.

Relationships

None of the previously described species was sequenced, therefore the differentiation of new species is based on morphological characters (see also Table 2), host and geographic distribution.

**Table 2:**
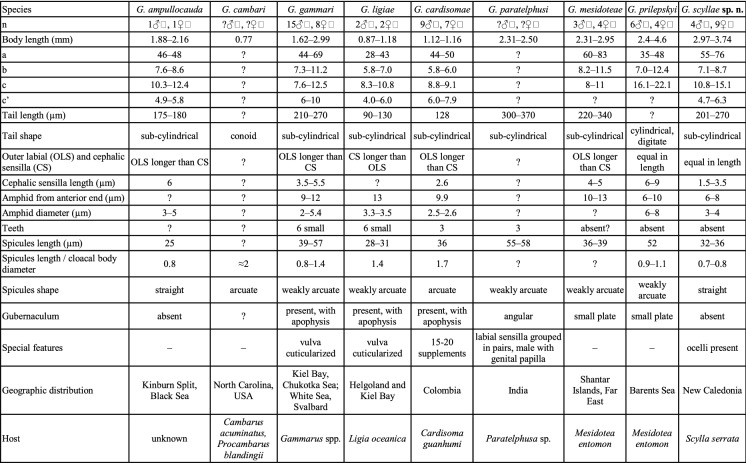
Comparison between different species of the genus *Gammarinema*

*Gammarinema ampullocauda* is smaller than *G. scyllae* **sp. n.** with body length of 1.88–2.16 mm compared to 2.97–3.74 mm in *G. scyllae* **sp. n.** The a-value of the *G. ampullocauda* is 46–48 and in *G. scyllae* **sp. n.** it is 55–76. The tail is shorter in *G. ampullocauda* (175–180 µm) than in *G. scyllae* **sp. n.** (201–270 µm). The length of cephalic sensilla is 6 µm in *G. ampullocauda* compared to 1.5–3.5 µm in *G. scyllae* **sp. n.** Spicules are shorter in *G. ampullocauda* (25 µm) than in *G. scyllae* sp. n. (32–36 µm). *G. ampullocauda* was found in Black Sea, its host is unknown.

*Gammarinema cambari* is described very superficially. The new species is much bigger than *G. cambari* with body length reaching 2.97–3.74 mm in new species compared to 0.77 mm in *G. cambari*. The shape of the tail differs between two species, the tail of *G. scyllae* **sp. n.** is sub-cylindrical and the tail of *G. cambari* is conoid. The spicules are arcuate in *G. cambari* but straight in *G. scyllae* **sp. n.** *G. cambari* was found in North Carolina in the USA and uses *Cambarus acuminatus* and *C. blandingii* as a host, while *G. scyllae* **sp. n.** was found in New Caledonia.

*Gammarinema scyllae* **sp. n.** is more than twice as big as *G. cardisomae* that has body length of 1.12–1.16 mm compared to 2.97–3.74 mm in *G. scyllae* **sp. n.** Moreover, *G. scyllae* **sp. n.** has higher values of many ratios than *G. cardisomae*: a-value is 44–50 in *G. scyllae* sp. n. compared to 55–76 in *G. cardisomae*, b-value is 5.8–6.0 in *G. scyllae* **sp. n.** compared to 7.1–8.7 in *G. cardisomae* and c-value is 8.8–9.1 in *G. scyllae* **sp. n.** compared to 10.8–15.1 in *G. cardisomae.* Exception is a c´-value where *G. cardisomae* has higher value of 6.0–7.9 compared to 4.7–6.3 in *G. scyllae* **sp. n.** The tails of the two species are the same in shape but differ in length: 128 µm in *G. cardisomae* and 201–270 µm in *G. scyllae* **sp. n.** *G. cardisomae* has three teeth while teeth are absent in *G. scyllae* **sp. n.** The spicules are different in shape in the two species, the spicules in *G. cardisomae* are arcuate and in *G. scyllae* sp. n. the spicules are straight. *G. cardisomae* has a gubernaculum with apophysis which is absent in the *G. scyllae* **sp. n.** *G. cardisomae* was found in Colombia, South America.

*Gammarinema scyllae* **sp. n.** is somewhat bigger than *G. gammari* with a body length ranging from 1.62–2.99 mm in the latter and 2.97–3.74 mm in the former. The a-, b- c- ratios are similar/overlapping, but the c´-value is different, 6–10 in the *G. gammari* compared to 4.7–6.3 in *G. scyllae* **sp. n.** The amphid is located 9–12 µm from the anterior end in *G. gammari* compared to 6–8 µm in *G. scyllae* **sp. n.** *G. gammari* has six small teeth while teeth are absent in the *G. scyllae* **sp. n.** *G. gammari* has slightly larger (39–57 µm) and weakly arcuate spicules while *G. scyllae* **sp. n.** has shorter (32–36 µm) and nearly straight spicules. *G. gammari* has a small plate-like gubernaculum, whereas gubernaculum is absent in *G. scyllae* **sp. n.** *G. gammari* uses multiple species of *Gammarus* as host and is found in Northern Europe.

*Gammarinema ligiae* is smaller with a body length of only 0.87–1.18 mm while *G. scyllae* **sp. n.** is 2.97–3.74 mm long. Body proportions are also different: a-value is 28–43 in *G. ligiae* and 55–76 in *G. scyllae* **sp. n.**, b-value is 5.8–7.0 in *G. ligiae* and 7.1–8.7 in *G. scyllae* **sp. n.**, c-value is 8.3–10.8 in *G. ligiae* and 10.8–15.1 in *G. scyllae* **sp. n.** The tail is shorter (90–130 µm) in *G. ligiae* than in *G. scyllae* **sp. n.** (201–270 µm)*.* The amphid in *G. ligiae* is positioned more posterior from the anterior end (13 µm) than in *G. scyllae* **sp. n.** (6–8 µm). *G. ligiae* has six small teeth that *G. scyllae* **sp. n.** lacks. The spicules are straight and slightly larger (32–36 µm) in *G. scyllae* **sp. n.** while they are weakly arcuate and smaller (28–31 µm) in *G. ligiae.* The gubernaculum is present and has apophysis in *G. ligiae* but is absent in *G. scyllae* **sp. n.** *G. ligiae* stands out from other species of the genus by having cuticularised vulva. *G. ligiae* is distributed in Northern Europe (Helgoland and Kiel Bay) and uses *Ligia oceanica* as a host.

*Gammarinema scyllae* **sp. n.** is bigger than *G. mesidoteae* with a body length of 2.97–3.74 mm compared to 2.31–2.95 mm in *G. mesidoteae*. The cephalic sensilla are slightly longer (4–5 µm) in *G. mesidoteae* than in *G. scyllae* **sp. n.** (1.5–3.5)*.* The amphid is positioned 10–13 µm from the anterior end in *G. mesidoteae* and 6–8 µm in *G. scyllae* **sp. n.** The spicules in *G. mesidoteae* are both longer 36–39 µm and have a different shape (weakly arcuate) compared to *G. scyllae* **sp. n.** that has 32–36 µm long straight spicules. *G. mesidoteae* has gubernaculum in the shape of a small plate while gubernaculum is completely absent in *G. scyllae* **sp. n.** *G. mesidoteae* lives in Far East and is known to have *Mesidotea entomon* as a host.

*Gammarinema paratelphusi* is described very superficially, with few measurements given, and with some unusual morphological features, such as cephalic sensilla arranged in pairs or pre- and postcloacal sensilla present in males. The new species can be easily separated from *G. paratelphusi* in having shorter (32–36 µm) and differently shaped (straight) spicules, which are weakly arcuate and 55–58 µm long in *G. paratelphusi*, absence of teeth in stoma (present in *G. paratelphusi*), absence of pre- and postcloacal sensilla in male (present in *G. paratelphusi*). *G. paratelphusi* was found in India in a freshwater crab *Paratelphusa* sp.

*Gammarinema prilepskyi* has 2.4 mm long body while *G. scyllae* **sp. n.** is 2.97–3.74 mm long. The a-value differs between the two species, 35–48 in *G. prilepskyi* and 55–76 in *G. scyllae* **sp. n.** Same with c-value which is 16.1–22.1 in *G. prilepskyi* and 10.8–15.1 in *G. scyllae* **sp. n.** The tail is cylindrical with digitate tip in *G. prilepskyi* and sub-cylindrical in *G. scyllae* **sp. n.** The cephalic sensilla are longer in *G. prilepskyi* (6–9 µm) than in *G. scyllae* **sp. n.** (1.5–3.5 µm). The length and shape of spicules are different with *G. prilepskyi* having weakly arcuate 52 µm long spicules, while the spicules in *G. scyllae* **sp. n.** are straight and 32–36 µm long. *G. prilepskyi* has small plate-like gubernaculum and *G. scyllae* **sp. n.** has none. The geographic distribution of *G. prilepskyi* is limited to the Barents Sea and the host is *Mesidotea entomon.*

Identification key to species of the genus* Gammarinema*

- 1. Ocelli present ... *G. scyllae ***sp. n.**
– Ocelli absent ... 2
- 2. Male with numerous (15–20) supplements ... *G. cardisomae*
– Male without supplements ... 3
- 3. Labial sensilla grouped in pairs ... *G. paratelphusi*
– Labial sensilla equidistantly arranged ... 4
- 4. Body shorter than 0.8 mm; tail conoid; spicules equal to two cloacal body diameters in length ... *G. cambari*
– Body longer than 0.8 mm; tail subcylindrical; spicules less than 1.5 cloacal body diameters in length... 5
- 5. Tail with distinct digitate distal part; outer labial and cephalic sensilla equal in length ... *G. prilepskyi*
– Tail without digitate distal part; outer labial and cephalic sensilla unequal in length... 6
- 6. Vulva distinctly cuticularized; gubernaculum with apophysis ... 7
– Vulva not cuticularized; gubernaculum plate-like or absent ... 8
- 7. Outer labial sensilla longer than cephalic sensilla; tail relatively long (210-270 µm; c’=6–10) ... *G. gammari*
– Cephalic sensilla longer than outer labial sensilla; tail relatively long (90-130 µm; c’=4–6) ... *G. ligiae*
- 8. Spicules 25 µm long ... *G. ampullocauda*
– Spicules 36–39 µm long ... *G. mesidoteae*

***Monhystrium ***Cobb, 1920

- *Type species:*
- *Monhystrium transitans *Cobb, 1920, by original designation.
- *Other species:*
- *Monhystrium wilsoni* (Baylis, 1915) Cobb, 1920
- = *Monhystera wilsoni *Baylis, 1915
- *Monhystrium inquilinus* Riemann, 1969
- *Monhystrium* *brevis *Yoshimura, 1990
- *Monhystrium* *tenuis *Yoshimura, 1990
- *Monhystrium mangrovi* **sp. n.**

*Genus diagnosis:* Body length medium to long (0.8–1.7 mm). Inner labial sensilla papilliform; outer labial and cephalic sensilla in one circle, same or different in length, with either outer labial or cephalic sensilla being longer than the others. Amphids round; less than 1.5 head diameters from the anterior body end. Ocelli present or absent. Buccal cavity cuticularized and distinctly divided into two chambers: anterior chamber conoid; posterior chamber spherical, with sharp tooth-like anterior edges, with or withour denticles. Pharynx cylindrical. Progaster present. Ventral gland present or absent, excretory pore opens along the anterior region of the pharynx. Ovary and testis on the right-hand side of intestine. Spicules simple and narrow, weakly arcuate. Gubernaculum platelike. Precloacal spine may be present. Bursa present. Paired subventral papilla present, pre- and postcloacal, within or outside bursa. Tail conoid to subcylindrical, digitate. Caudal glands opening in via common spinneret. Usually found in gill chambers of crustaceans.

*Note:* The genera *Monhystrium* and *Diplolaimelloides* Meyl, 1954 are close morphologically, with *Diplolaimelloides delyi* Andrássy, 1958 being also found in the gill chambers of land crabs, the only clear morphological difference between them is the more strongly developed and cuticularized posterior stoma chamber with inward-pointing tooth-like anterior edges in *Monhystrium.* Sequenced species from two genera do not form a monophyletic lineage (Figure 7), however, the limited taxon sampling seriously undermines our understanding of the phylogeny of this group in general.

***Monhystrium mangrovi ***sp. n.

*Type host and locality:* The nematodes were found in the gill chambers of the mangrove crab *Neosarmatium* sp. collected near Poya, Poya commune, west coast of the Northern Province, New Caledonia (HYNC4625; July 3, 2018).

*Type material:* Holotype male and eleven juvenile paratypes on slides MNHN-BN512 and MNHN-BN13 are deposited in the meiofauna collection of the National Museum of Natural History in Paris, France (MNHN). Three females and 12 male paratypes on slides SMNH Type-9351 and SMNH Type-9352 are deposited in the Invertebrate type collection of the Department of Zoology, Swedish Museum of Natural History, Stockholm, Sweden.

*Etymology*: The species name *mangrovi* refers to the host of this species, a species of a mangrove crab.

*ZooBank registration*: urn:lsid:zoobank.org:pub:A0F29FA2-B06B-4CFF-A100-BE4F292E8188 (publication); urn:lsid:zoobank.org:act:B8A75B36-6BB5-4C72-88B2-EBDC03B6AC5D (species).

*GenBank acc. numbers:* Sequences obtained are deposited in GenBank under the accession numbers MZ274177 and MZ274178 for the D2-D3 segment of the 28S rRNA gene and MZ274173 and MZ274174 for the nearly full-length 18S rRNA gene.

### Description

*Diagnosis: Monhystrium mangrovi* **sp. n.** is characterised by 1–1.4 mm long body; outer labial sensilla longer than cephalic sensilla, amphid located at level with posterior stoma chamber, denticles in posterior stoma chamber and five pairs of genital papilla on tail. In gill chambers and body cavity of mangrove crab *Neosarmatium* sp.

**Fig. 4:**
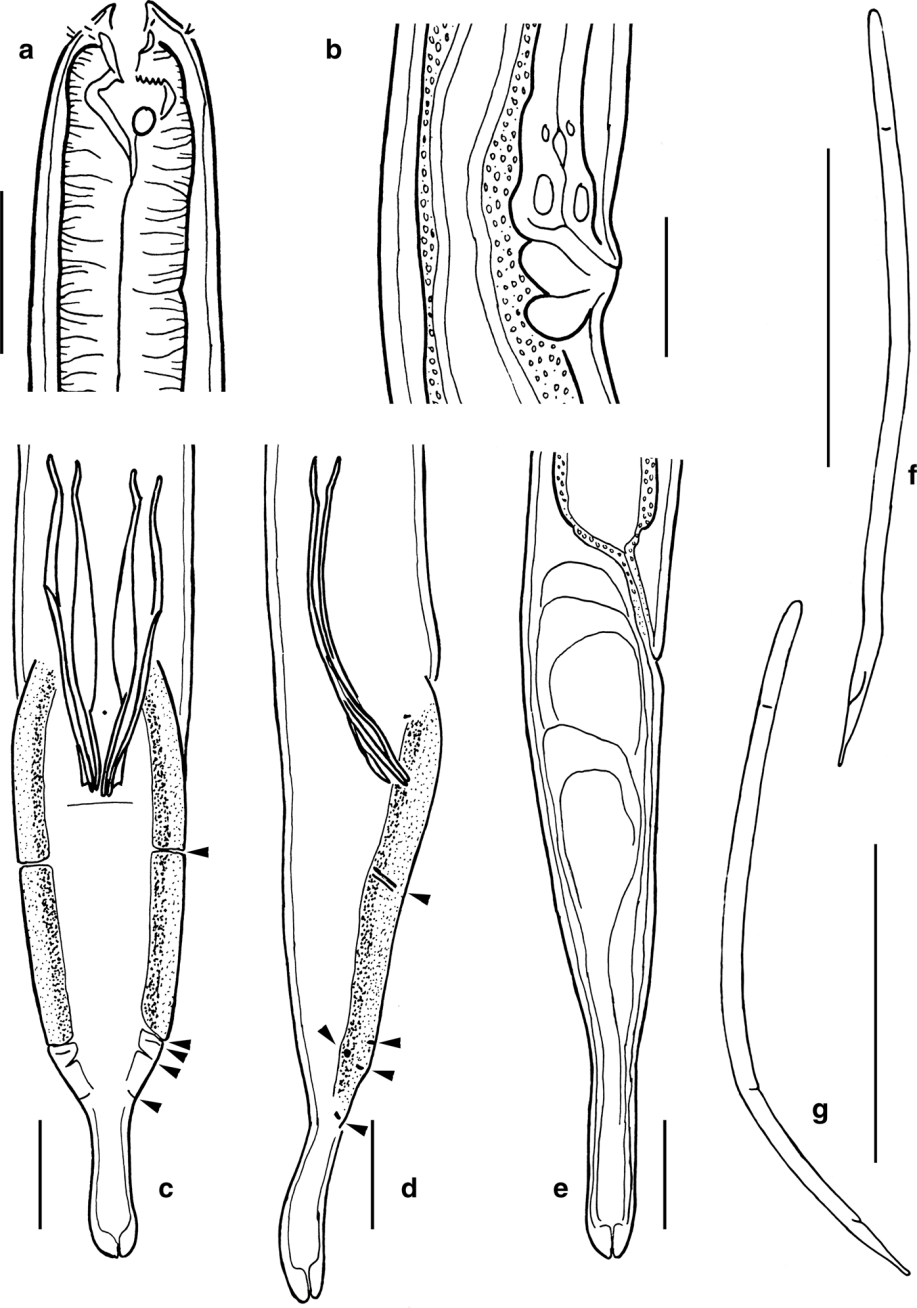
*Monhystrium mangrovi* **sp. n.** a. Anterior end (combined view); b. Vulval region; c. Male posterior end, ventral view (arrowheads point to genital papilla); d. Male posterior end, lateral view (arrowheads point to genital papilla); e. Female tail; f. Entire male; g. Entire female. Scale bars: a–e = 20 µm, f–g = 500 µm.

**Fig. 5:**
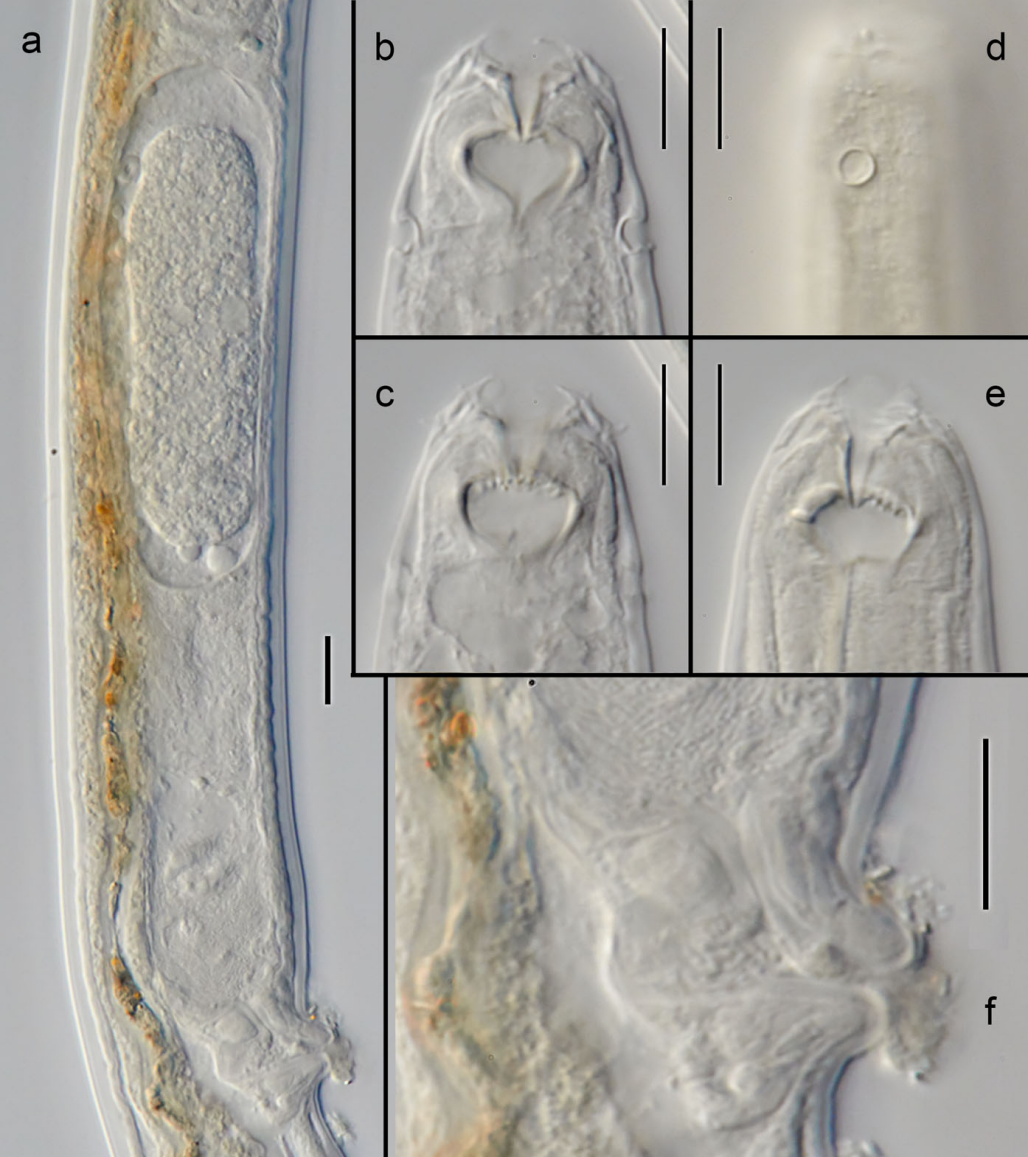
*Monhystrium mangrovi* **sp. n.** a. Vulval region and uterus; b–c. Anterior end, dorso-ventral view at different optical planes; d. Amphid; e. Anterior end, lateral view (ventral side to the right), median optical section; f. Vulva. Scale bars: a–e = 10 µm.

**Fig. 6:**
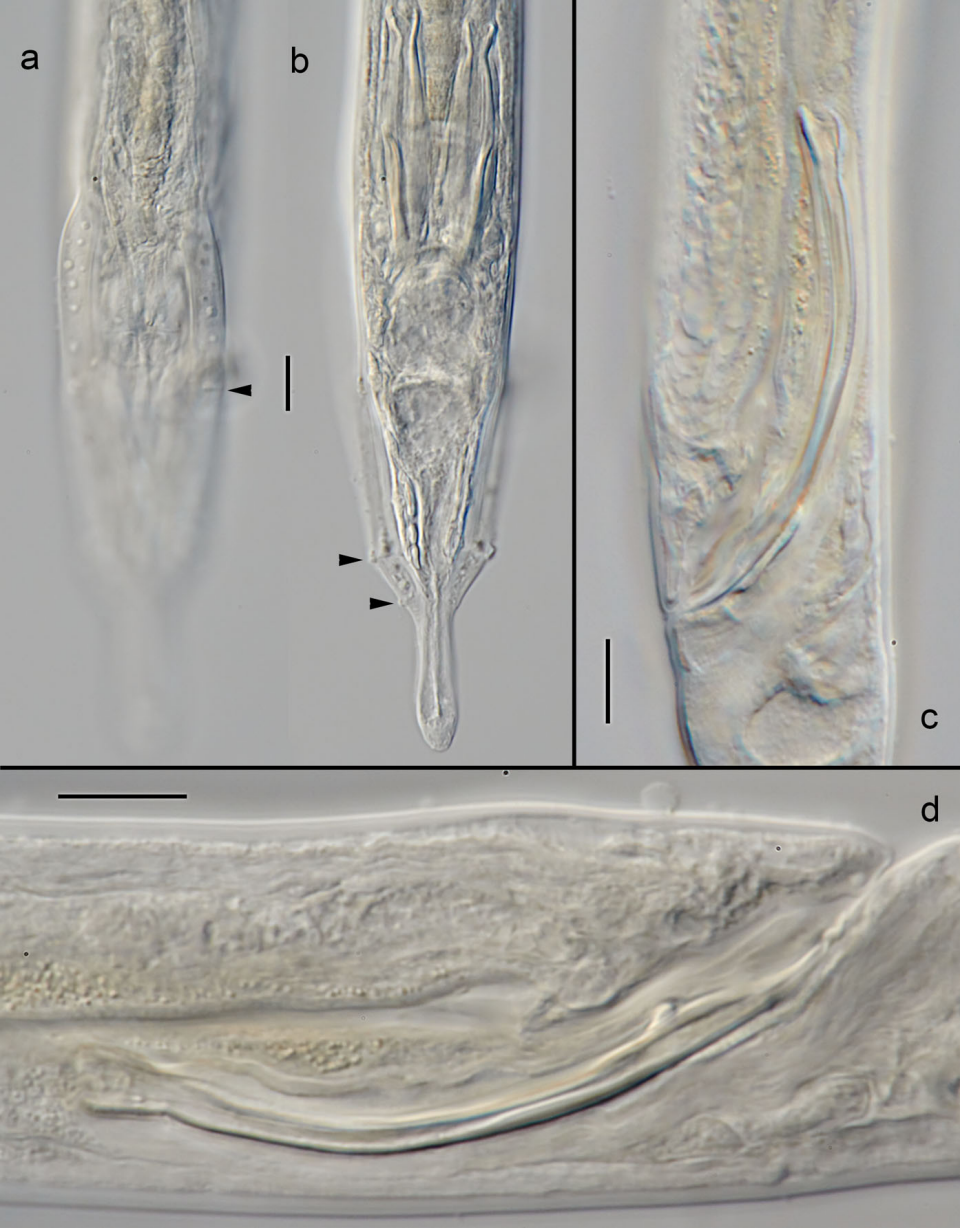
*Monhystrium mangrovi* **sp. n.** Male. a–b. Posterior end, ventral view at different focal planes showing anterior and posterior part of bursa; c–d. Spicules. Scale bars: a–e = 10 µm.

*Adult.* (Figures 4, 5, 6, Table 3). Body cylindrical, nearly straight upon fixation, tapering slightly towards both extremities along pharyngeal region and on tail. Cuticle smooth. Somatic sensilla present, small setiform. Body pores absent. Lateral alae absent. Cephalic region rounded. Six equal lips surrounding mouth opening. Inner labial sensilla papilliform, located on anterior surface of lips. Outer labial sensilla setiform, located at the base of lip region. Cephalic sensilla small papilliform, located at the same level as outer labial sensilla. Amphideal opening round, located at level of posterior stoma chamber. Ocelli absent. Buccal cavity composed of two chambers. Anterior chamber of buccal cavity conical. Posterior chamber broad spherical with multiple teeth on the anterior part of its ventral surface. Pharynx uniformly muscularized along its entire length, gradually widening posteriorly but without any valves or bulbs. Cardia small. Intestinal lumen well developed, progaster present. Secretory-excretory system and secretory-excretory pore not visible, absent. Tail conical, with digitiform terminus. Caudal glands present, opening towards exterior via a common spinneret. Caudal gland cells located in the tail. Spinneret not cuticularised.

**Table 3:** Measurements of *Monhystrium mangrovi* **sp. n.** All measurements in µm

Characters	holotype male	13 males	4 females
Body length	1225	1260 ± 84 (1087–1366)	1213 ± 13 (1197–1225)
Body diameter	31	32 ± 42 (28–35)	35 ± 1 (33–36)
Pharynx length	173	177 ± 7 (162–185)	168 ± 14 (151–182)
Tail length	92	91 ± 7 (79–100)	118 ± 8 (112–129)
Anal body diameter	23	28 ± 3 (23–32)	25 ± 2 (23–26)
a	39.8	39.4 ± 2.9 (33.3–43.2)	35.0 ± 1.6 (33.4–37.0)
b	7.1	7.1 ± 0.4 (6.4–7.8)	7.3 ± 0.6 (6.7–8.0)
c	13.3	13.9 ± 1.3 (12.2–16.1)	10.3 ± 0.6 (9.5–10.8)
c´	3.0	2.8 ± 0.3 (2.2–3.4)	4.8 ± 0.4 (4.3–5.2)
V	–	–	66.6 ± 1.3 (65.1–68.0)
Labial region diameter	12	12.2 ± 1.4 (10.5–14.0)	12.5–14.0
Cephalic setae length	1.5	1.5–2.0	2.0
Amphid diameter	3	3.0–3.5	3.0–3.5
Amphid from anterior end	7.5	12.0 ± 3.4 (7.5–18.5)	13.1 ± 2.8 (10.5–16.0)
Stoma length	15	15.4 ± 0.9 (14.5–16.5)	17.0 ± 0.8 (16.5–18.0)
Vulva length	–	–	17.8 ± 2.7 (14.5–20.0)
Rectum length	–	–	27.6 ± 3.3 (24.0–32.0)
Spicule length	66	67 ± 3 (62–73)	–
Bursa length	27	29.3 ± 4.6 (25.5–41.0)	–

*Female*. Reproductive system monodelphic. Ovary branch outstretched, extends anterior but not reaching the cardia, on the right-hand side of intestine. Post-vulval uterine sac absent. Vagina directed anteriorly. Vulva located posterior to midbody, a transverse ventral slit, not cuticularised.

*Male.* Reproductive system monorchic, on the right-hand side of intestine. Spicules paired and symmetrical, arcuate, very long, curved ventral, with angular manubrium and cylindrical shaft, thin velum and lateral projections near their tips equal to 2.1–3.0 anal body diameters in length. Gubernaculum plate-like. No midventral pre- or post- cloacal supplements. Precloacal spine present, short distance in front of cloacal opening. Bursa well-developed, starts anterior to cloaca and extends to the posterior third of tail. Five pairs of genital papillae present: one pair just posterior to cloaca, three pairs near posterior end of bursa and one pair just behind bursa

*rRNA.* Sequences include two nearly full length 18S rRNA gene and two partial 28S rRNA gene representing D2D3 domain. Sequence variability of 18S rRNA gene was small, less than 1–3 bases difference; sequences of D2D3 domain of 28S rRNA gene were identical. This is the first species from the genus *Monhystrium* to be sequenced.

Relationships

None of the previously described species was sequenced, therefore the differentiation of the new species is based on morphological characters (see also Table 4), host and geographic distribution. The new species can be separated from all other known species of the genus by the presence of denticles in the posterior stoma chamber.

**Table 4:**
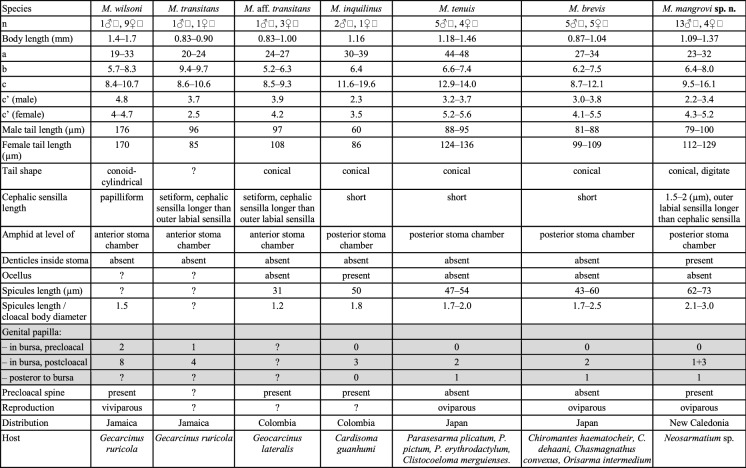
Comparison between different species of the genus *Monhystrium*

*Monhystrium mangrovi* **sp. n.** is smaller than *M. wilsoni* with body length of 1.09–1.37 mm compared to 1.4–1.7 mm in *M. wilsoni*. *M. wilsoni* has longer tail (176 µm) with c´-value of 4.8 compared to 79–129 µm in *M. mangrovi* **sp. n.** with c´-value of 2.2–3.4. The position of the amphid is different: at level with anterior stroma chamber in *M. wilsoni* compared to at level with posterior stoma chamber in *M. mangrovi* **sp. n.** There are differences in the number of genital papilla in both species: *M. wilsoni* has two pairs of precloacal and eight pairs of postcloacal genital papilla within bursa, while *M. mangrovi* **sp. n.** have no precloacal papilla and four pairs of postcloacal papilla in bursa arranged in two groups (1+3). Precloacal spine is present in *M. wilsoni* but absent in *M. mangrovi* **sp. n.** The mode of reproduction is viviparous in *M. wilsoni* and oviparous in *M. mangrovi* **sp. n.** *M. wilsoni* is found in Jamaica.

*Monhystrium mangrovi* **sp. n.** is bigger than *M. transitans* with body length of 1.09–1.37 mm compared to 0.83–0.90 mm in *M. transitans*. *M. transitans* has relatively shorter pharynx (b-value 9.4–9.7) compared to *M. mangrovi* **sp. n.** (b-value 6.4-8.0). In *M. transitans* the amphid is located at level with the anterior stoma chamber and in *M. mangrovi* **sp. n.** at level with posterior stoma chamber. The *M. transitans* has one pair of precloacal genital papilla and four pairs of postcloacal genital papilla within bursa while *M. mangrovi* **sp. n.** has no precloacal genital papilla in bursa and four pairs of postcloacal papilla arranged in two groups (1+3) in bursa. The known distribution of *M. transitans* is limited to Jamaica*.*

*Monhystrium* aff. *transitans* is smaller than *M. mangrovi* **sp. n.** with a body length of 0.83–1.0 mm compared to 1.09–1.37 mm in *M. mangrovi* **sp. n.** The amphid is located at level with anterior stoma chamber in *M.* aff. *transitans* but at level with posterior stoma chamber in *M. mangrovi* **sp. n.** The spicules are much shorter in *M.* aff. *transitans* (31 µm) than in *M. mangrovi* sp. n. (62–73 µm). Precloacal spine is present in *M.* aff. *transitans* but absent in *M. mangrovi* **sp. n.** Geographic distribution of *M.* aff. *transitans* is limited to Colombia.

*Monhystrium inquilinus* has shorter tail both in males 60 µm (compared to 79–100 µm in *M. mangrovi* **sp. n.**) and in females 86 µm (compared to 112–129 µm in *M. mangrovi* **sp. n.**). Spicules are shorter in *M. inquilinus* (50 µm) compared to 62–73 µm in the new species. Only three pairs of genital papilla are present postcloacal in bursa in *M. inquilinus* but in *M. mangrovi* **sp. n.** there are four pairs of postcloacal papillae arranged in two groups (1+3) in bursa. Precloacal spine, and ocelli are present in *M. inquilinus* but both structures are absent in *M. mangrovi* **sp. n.** *M. inquilinus* is found in Colombia so far.

*Monhystrium tenuis* is slimmer than the new species, with a-value being higher (44–48) comparing to *M. mangrovi* **sp. n.** (23–32). The length of the spicules is different between species, 47–54 µm in *M. tenuis* and 62–73 µm in the *M. mangrovi* **sp. n.** There are only two pairs of genital papillae postcloacal in bursa in *M. tenuis* while *M. mangrovi* **sp. n.** has four pairs of postcloacal genital papilla arranged in two groups (1+3) in bursa. *M. tenuis* is found in Japan*.*

*Monhystrium brevis* is smaller with a body length of 0.87–1.04 mm compared to 1.09–1.37 mm in *M. mangrovi* **sp. n.** The length of the spicules smaller in *M. brevis* (43–60 µm) than in *M. mangrovi* **sp. n.** (62–73 µm). Another thing that separates the two species is that *M. mangrovi* **sp. n.** has four pairs of postcloacal genital papilla arranged in two groups (1+3) in bursa and *M. brevis* has only two pairs. *M. brevis* is found in Japan.

**Identification key to species of the genus** *Monhystrium*

- 1. Posterior stoma chamber with numerous denticles ... *M. mangrovi ***sp. n.**
– Posterior stoma chamber without denticles ... 2
- 2. Amphid at the level with anterior stoma chamber ... 3
– Amphid at the level with posterior stoma chamber ... 4
- 3. Cephalic sensilla papilliform; body 1.4–1.7 mm long ... *M. wilsoni*
– Cephalic sensilla setiform; body 0.8–1.0 mm long ... *M. transitans*
- 4. Ocelli present; precloacal spine present ... *M. inquilinus*
– Ocelli absent; precloacal spine absent ... 5
- 5. Body 1.2–1.5 mm long; a > 40; c > 12 ... *M. tenuis*
– Body 0.9–1.1 mm long; a < 40; c < 12 ... *M. brevis*

**Phylogenetic position of ***Gammarinema* and *Monhystrium*

**Fig. 7:**
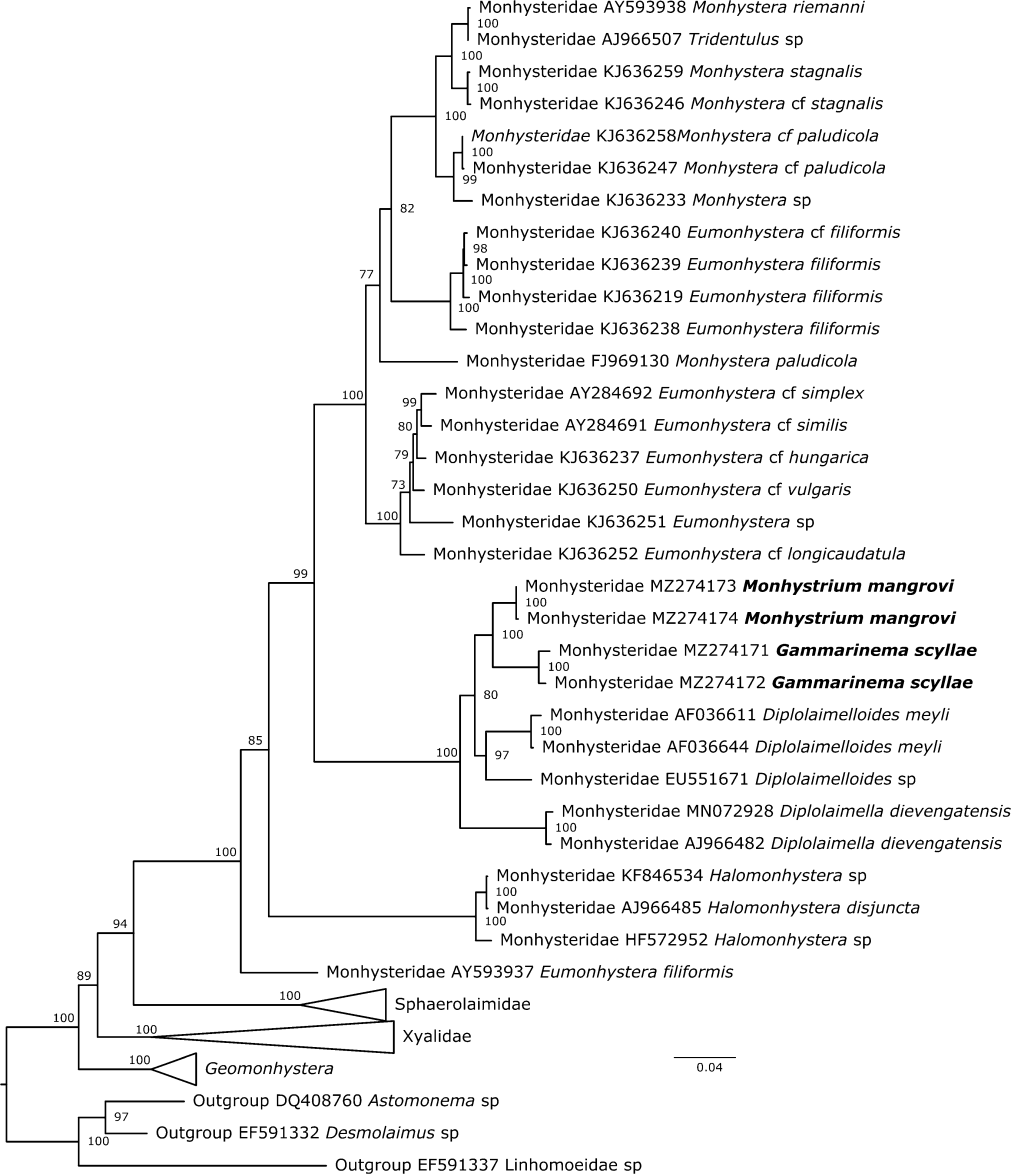
Phylogenetic position of *Gammarinema scyllae* **sp. n.** and *Monhystrium mangrovi* **sp. n.** based on 18S rDNA.

**Fig. 8:**
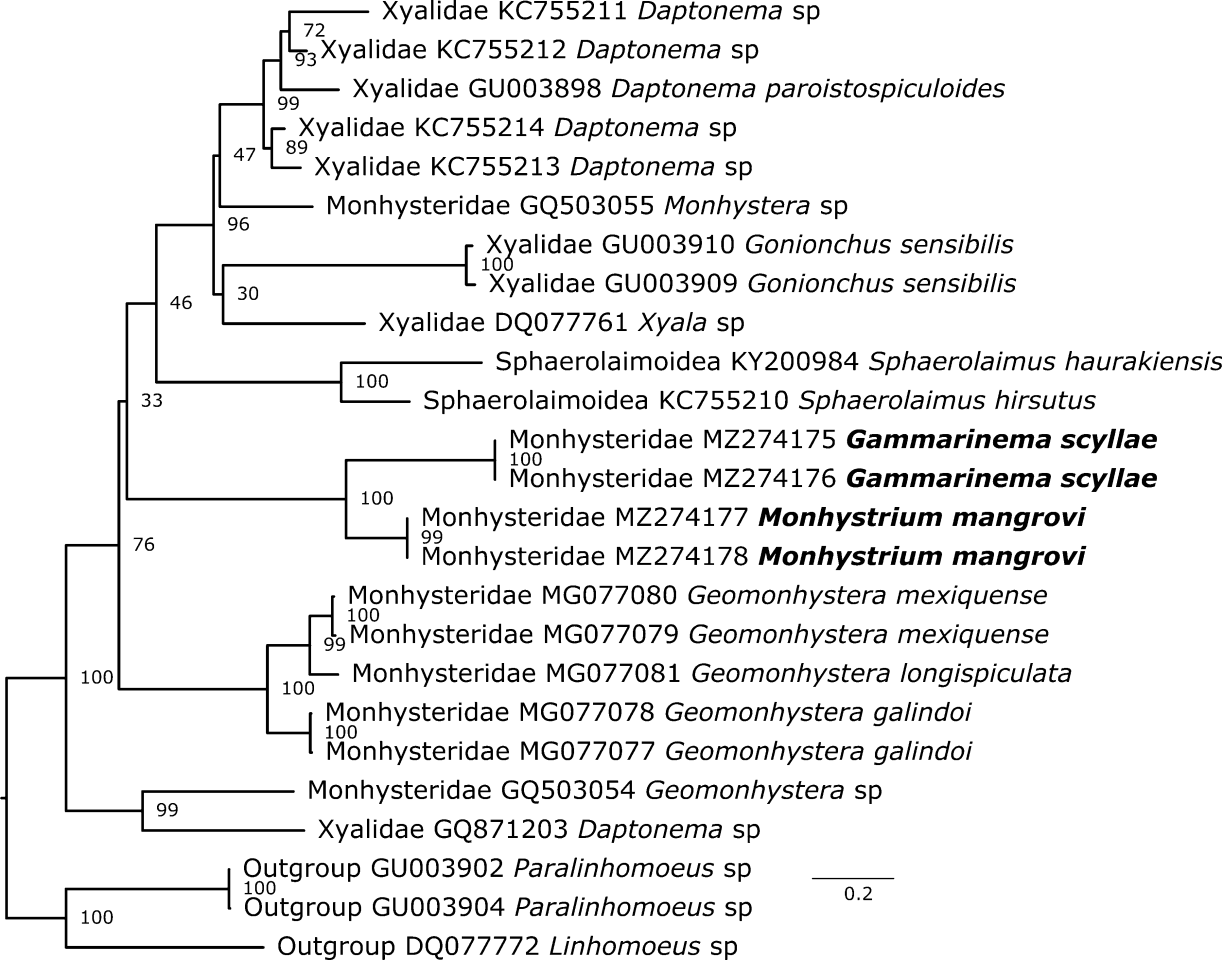
Phylogenetic position of *Gammarinema scyllae* **sp. n.** and *Monhystrium mangrovi* **sp. n.** based on partial 28S rDNA.

Two genes were used to build two different phylogenetic trees, 18S (Fig. 7) and 28S rDNA (Fig. 8), with members of the family Linhomoeidae being used as outgroups. The phylogeny based on 18S rDNA was the one most reliable with reasonably high bootstrap support values overall. It also covered a broader taxonomic diversity. The 28S rDNA tree included fewer taxa and showed lower bootstrap support for many clades. Both trees suggest *Gammarinema scyllae* **sp. n.** is most closely related to *Monhystrium mangrovi* **sp. n.**, both belonging to the family Monhysteridae. Current phylogeny indicates that both genera, *Gammarinema* and *Monhystrium* originate from a recent common ancestor. *Diplolaimella* and *Diplolaimelloides* were recovered as the closest relatives to the two genera and the four form a very well supported clade, in agreement with morphology-based theories. Moreover, the fact that all known species of *Monhystrium* are found exclusively in gill chambers of different land crabs and eight out of nine known species of *Gammarinema* are associated with crustaceans suggests that the common ancestor was also associated with crustaceans. Future studies should focus on sequencing other species from *Gammarinema, Monhystrium* and also of closely related commensalistic (*Odontobius, Tripylium, Diplolaimelloides delyi*) and free-living species to better understand the phylogeny of this group. Although the phylogeny did not support the subfamilies Diplolaimellinae and Monhysterinae, the family Monhysteridae received maximal support.

## Data Availability

All studied specimens are deposited in permanent and accessible repositories: National Museum of Natural History in Paris, France and Swedish Museum of Natural History, Stockholm, Sweden. Sequences are deposited in GenBank.
